# Biomechanical Forces in Prostate Cancer: Current Insights and Future Directions

**DOI:** 10.3390/cancers18040608

**Published:** 2026-02-12

**Authors:** Yunjie Ju, Dong Ni, Shimin Zou, Ping Dai, Jianhu Xie, Kangnan He, Yarong Song, Yifei Xing, Liang Chen

**Affiliations:** 1 Department of Urology, Union Hospital, Tongji Medical College, Huazhong University of Science and Technology, Wuhan 430022, China; 2 Department of Urology, The Third Affiliated Hospital of Shenzhen University, Shenzhen University, Shenzhen 518000, China

**Keywords:** prostate cancer, tumor biomechanics, mechanotransduction, extracellular matrix remodeling, tissue stiffness, solid stress, fluid shear stress

## Abstract

Over the past decade, research in tumor biomechanics has increasingly shown that cancer cells adapt to changing physical microenvironments by rewiring adhesion, cytoskeletal organization, and force-responsive signaling pathways, thereby shaping survival, invasion, and responses to therapy. Prostate cancer (PCa), like other solid tumors, resides in a highly dynamic mechanical milieu molded by extracellular matrix (ECM) remodeling, solid stress, and fluid shear forces. Available evidence generally supports that malignant prostate tissue is stiffer than benign tissue. During metastatic progression, however, the mechanical phenotype of PCa cells appears to undergo context-dependent remodeling. Such mechanical adaptations may help tumor cells withstand the physical challenges associated with circulation, adhesion switching, and colonization, and may intersect with the development of therapy resistance. Here, we synthesize recent advances in PCa biomechanics, highlight the intricate interplay between mechanical cues and tumor biology, and discuss opportunities to incorporate a mechanical perspective into diagnostic and therapeutic strategies. A deeper understanding of these processes may ultimately enable the development of emerging “mechanotherapies” for prostate cancer.

## Simple Summary

Prostate cancer evolves within a mechanically dynamic microenvironment shaped by extracellular matrix remodeling, tissue stiffness, solid stress, and fluid flow. Increasing evidence indicates that these biomechanical cues regulate tumor cell survival, invasion, metastatic colonization, and therapeutic response through mechanotransduction pathways. In this review, we summarize recent advances in prostate cancer biomechanics and discuss how integrating mechanical insights may improve diagnosis, risk stratification, and treatment strategies. Understanding force-related vulnerabilities may also facilitate the development of emerging “mechanotherapies” for prostate cancer.

## 1. Introduction

Over the past decade, studies in tumor biomechanics have made it clear that the physical microenvironment is more than a passive backdrop; it is an active set of cues that cancer cells can detect and convert into signaling outputs, with consequences for proliferation, invasion, metastasis, and treatment response [1,2]. Among them, prostate cancer (PCa) resides in a highly dynamic mechanical ecosystem in which tumor-driven volumetric expansion caused by aberrant ECM deposition/remodeling, mechanical confinement from the prostatic capsule and surrounding stroma, and abnormal vasculature with interstitial fluid flow together generate a complex stress landscape characterized by coexisting solid stress/compressive forces, alterations in matrix stiffness, and fluid-related mechanical cues [3,4]. These cues may remodel the tumor microenvironment via vascular compression, hypoxia-associated metabolic shifts, and changes in immune infiltration and drug delivery, while also directly reprogramming tumor-cell adhesion, cytoskeletal tension, and mechanoresponsive transcription. Importantly, PCa cell mechanical properties seem to be context-dependent and may differ across metastatic stages [5]. Here, we synthesize current evidence on solid stress/compression, ECM remodeling and matrix stiffening, and fluid pressure/shear stress in PCa, highlight the mechanosensing and mechanotransduction pathways that link these cues to cellular behaviors, and discuss the translational rationale for pairing mechanical interventions with standard therapies.

### 1.1. Classification and Functional Roles of Biomechanical Cues in Prostate Cancer

**Figure 1:**
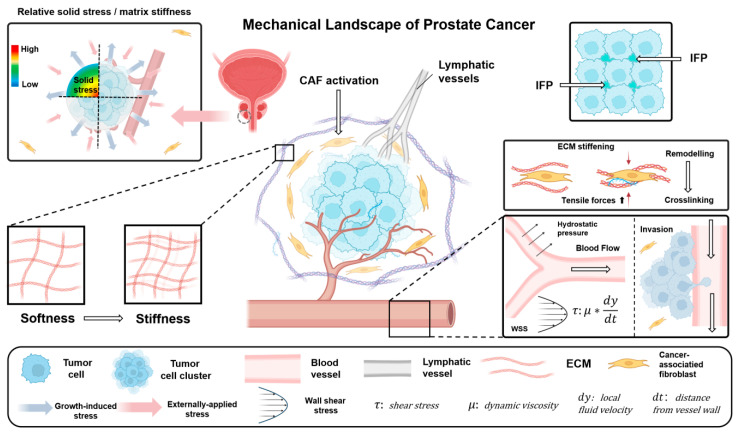
Schematic illustration of the multiscale mechanical microenvironment in prostate cancer. ECM deposition, remodeling, and crosslinking progressively stiffen the matrix and increase solid stress, which can be further augmented by CAFs. Solid stress compresses blood and lymphatic vessels, reducing perfusion and increasing vascular permeability, thereby raising IFP. In parallel, tumor cells and clusters experience hydrostatic pressure and fluid forces, including wall shear stress (WSS) in vessels and shear generated by interstitial flow in the stroma.

Prostate cancer cells in tumors are constantly influenced by various biomechanical forces. As shown in Figure 1, these mechanical factors arise from different levels and superimpose on each other to ultimately form an integrated mechanical environment. In the following sections, we summarize several common mechanical stimuli in PCa and outline their functional roles in tumor initiation and progression.

### 1.2. Solid Stress and Compressive Forces

Direct studies on solid stress in prostate cancer are still relatively limited, but similar to other solid tumors, compressive stress within the tumor can accumulate markedly. Solid stress can be summarized into two categories: growth-induced stress, which arises from the continuous expansion of the solid-phase volume of tumor cells and the solid ECM; and externally applied stress, which comes from mechanical confinement of tumor expansion by the prostatic capsule and surrounding stroma [6,7]. The superposition of these two stresses creates a typical mechanical gradient with compressive loads in which the tumor center is under high compression while the periphery experiences tensile stress. This structure of internal high pressure and external confinement can drive tumor cells to migrate along directions of lower stress, thereby promoting local invasion and distant metastasis as a means to relieve the increased pressure in the tumor microenvironment [8]. Based on shear-wave elastography and other biomechanical measurements, the Young’s modulus of prostate cancer tissue is markedly higher than that of benign prostatic hyperplasia or normal prostate tissue, indicating greater tissue stiffness and lower compressibility [9,10,11]. This finding suggests sustained accumulation of solid stress in prostate cancer. In addition, the prostatic capsule and surrounding stroma provide substantial mechanical confinement, such that the growing tumor experiences pronounced outward counter-pressure during expansion, further aggravating intratumoral compressive stress. Under prolonged high-pressure conditions, local capillaries can be compressed and collapse, perfusion becomes limited, and a hypoxic microenvironment develops, which in turn promotes increased invasiveness and metabolic reprogramming in tumor cells.

Cancer-associated fibroblasts (CAFs) in the prostate cancer microenvironment are a major active, cell-derived source of solid stress [12]. Mechanical strain arising from tumor expansion and ECM remodeling can activate CAFs, enabling them to generate traction forces through the actomyosin–nonmuscle myosin II (actomyosin–NMII) contractile system and to continuously synthesize and reorganize ECM components such as collagen and fibronectin [13,14,15], and these processes may promote the formation and maintenance of cell-derived solid stress at the tissue scale and, in specific contexts, provide mechanical conditions that facilitate spatial reorganization and migration of tumor cells. CAF-driven contraction and matrix remodeling can further promote ECM stiffening and fibrosis and, by compressing vessels, restricting drug penetration, and limiting immune-cell entry into the tumor core, contribute to a “mechanical barrier” microenvironment [13,16,17].

Taken together, solid stress can be viewed as an important physical hallmark of the interplay between structural remodeling and biological evolution in prostate cancer, and it may contribute to shaping the mechanical adaptation, invasive phenotypes, and therapeutic responses of tumor cells.

### 1.3. ECM Remodeling and Changes in Matrix Stiffness

ECM stiffness (also referred to as matrix stiffness) dynamically evolves during tumor progression and can influence cancer cell behavior across multiple dimensions, including morphology, proliferation, sensitivity to apoptosis, migration/invasion, differentiation, and anchorage dependence [18,19,20]. In general, biochemical alterations in the ECM and changes in its mechanical properties tend to occur in parallel and reinforce each other, collectively driving the tumor microenvironment in many solid cancers from a relatively homeostatic architecture toward a more stiffened and mechanically constrained state [15]. In prostate cancer, available evidence likewise suggests that a similar trajectory of matrix remodeling and stiffening may contribute to disease progression [21,22,23].

Tumor-associated ECM is composed of structural proteins such as collagens, regulatory molecules, and diverse accessory/secreted components that provide essential mechanical and biochemical cues to cells [24,25]. Among these, type I collagen is typically regarded as one of the principal load-bearing elements, while other proteins can also markedly shape matrix mechanics by regulating ECM production, crosslinking, and degradation [26,27,28]. Multiple studies indicate that increased collagen deposition together with enhanced fiber alignment/organization is closely associated with tissue stiffening and may be accompanied by the acquisition of migration- and metastasis-related phenotypes [29,30].

Notably, signs of ECM remodeling emerge early in PCa. During prostatic intraepithelial neoplasia (PIN), activated fibroblasts surrounding acini display a stronger capacity for type I collagen production, which has been considered an important event in early adenocarcinoma evolution [31,32]. With disease progression, collagen further accumulates within tumor regions, and its abundance shows a significant positive correlation with Gleason score [33]. In high-grade prostate cancer, collagen fibers are often denser and more highly aligned, and their spatial organization is closely associated with increased invasiveness [34].

During ECM remodeling, collagen is not the only component that changes. Distinct laminin subtypes also exhibit dynamic alterations. Study shows laminin-332 expression is markedly reduced during PCa progression, whereas laminin-511 appears relatively preserved [35,36]. As adhesive substrates, laminins can directly modulate tumor cell migratory capacity [37]. For instance, laminin-421 can cooperate with type I/III collagen and fibronectin to enhance the migratory activity of PCa cells [38].

Additional components likewise contribute to matrix stiffening and signaling reprogramming. Tenascin-C is frequently upregulated in the PCa stroma, and its high expression is closely associated with lymph node metastasis and poor overall survival [39]. Osteopontin is persistently elevated in CRPC and bone metastasis models and is considered one of the molecular markers related to advanced disease and osteotropic phenotypes [40]. Aberrant deposition, crosslinking, and reorganization of these structural and regulatory proteins not only reshape ECM composition but also directly sculpt local mechanical properties, rendering the matrix stiffer and more restrictive.

Overall, sustained ECM remodeling and stiffening constitute key histological and structural basis of malignant progression in PCa, and these changes also reshape the local mechanical stress landscape and may sustain mechanotransduction pathway activation.

### 1.4. Interstitial Fluid Mechanics: IFP, Interstitial Flow, and WSS

Fluid pressure and interstitial fluid flow constitute key components of the mechanical microenvironment within tumors [41]. In the abnormal neovasculature of tumors, increased vascular permeability promotes fluid extravasation; meanwhile, impaired tumor-associated lymphatic drainage leads to persistent interstitial fluid accumulation and a sustained elevation of interstitial fluid pressure (IFP). At the same time, the strong hydrophilicity of proteoglycan components in the tumor ECM further increases tissue water content, making local pressure more difficult to relieve. When a pressure difference forms between the tumor core and the periphery, a convective tendency from the center toward the edge may arise within the tumor. Under certain conditions, this convection occurs along interstitial paths with lower resistance, promoting the transport of growth factors and chemokines, and therapeutic molecules along preferential flow paths, thereby reshaping extracellular concentration gradients and influencing drug delivery and distribution within tumor tissue [42,43,44,45,46]. Within this “fluid–pressure” landscape, tumor and stromal cells can sense shear and tensile cues through mechanosensitive modules including integrin–FAK, cadherin/VEGFR complex, and Piezo/TRP channels, promoting adhesion reprogramming and migration [47,48,49,50,51,52]. These effects may further synergize with ECM fiber orientation. Notably, elevated IFP and microvascular compression are accompanied by reduced perfusion and aggravated hypoxia, which can enhance invasive and metabolic reprogramming while limiting convective entry and intratumoral diffusion of chemotherapeutic agents [53,54].

In addition to interstitial-level convection and pressure loading, tumor cells are also exposed to shear stimuli generated by fluid flow when interacting with the vascular or lymphatic system, particularly during adhesion and transendothelial migration. In mechanical terms, fluid shear stress (FSS) is an umbrella concept referring to shear forces arising from fluid flow, whereas wall shear stress (WSS) denotes the specific component acting on the endothelial wall of blood or lymphatic vessels. To avoid conceptual ambiguity, the terms “CTC-experienced FSS” or “circulatory FSS” are commonly used when referring to shear exposure of circulating tumor cells, in contrast to endothelial WSS.

During tumor cell adhesion and transendothelial migration, fluid shear stress generally exerts a biphasic influence on tumor cell–endothelium interactions: as shear magnitude increases, adhesion efficiency can initially rise and subsequently decline [55]. Moreover, the adhesion–rolling–arrest behaviors of single circulating tumor cells and multicellular clusters respond to FSS in distinct ways. Mechanistically, moderate FSS can induce reactive oxygen species (ROS) production and promote lamellipodia/filopodia formation, thereby facilitating transendothelial migration, whereas excessive shear tends to suppress cell arrest and transmigration [56]. In PCa models, shear stimulation has been shown to upregulate the cancer-driving complexes mTORC1/mTORC2, accompanied by increased endosomal, lysosomal and proton-pump activity and a more invasive phenotype [57]. On the other hand, FSS can create a therapeutic window of vulnerability: when PCa cells are exposed to shear, they become sensitized to TRAIL-induced apoptosis in a magnitude- and time-dependent manner, such that FSS within a certain range amplifies TRAIL-mediated cytotoxicity [58].

Beyond signaling rewiring, elevated shear can impose a direct biophysical selection pressure on circulating cells by challenging plasma-membrane integrity and repair capacity [55,59,60,61]. At high amplitudes, FSS can drive rapid deformation with transient membrane poration, increasing permeability and promoting cell death when damage accumulates [62,63]. Notably, prostate cancer cell lines differ in their tolerance to these insults, indicating that shear resistance is not uniformly conserved across metastatic PCa phenotypes [55,64]. In this setting, post-shear viability is closely linked to the efficiency of membrane repair, which may influence subsequent arrest and seeding competence. Consistent with this, greater cellular stiffness correlates with improved tolerance to shear-induced injury, whereas experimentally softening otherwise shear-tolerant cells reduces survival under elevated FSS [58,65]. Together, these findings point to a mechanical trade-off during dissemination, balancing deformability needed for navigating constricted spaces and extravasation with sufficient resistance and repair competency to endure circulatory shear.

In perfusion bioreactor systems that better mimic interstitial flow, low-magnitude, lymph-like WSS appears to be particularly important for PCa migration. Compared with static culture, physiological low flow (e.g., 0.05 mL·min^−1^) not only better supports PC3 cell survival but also markedly enhances transwell migration, whereas higher flow (e.g., 0.2 mL·min^−1^) triggers apoptosis through an imbalance between increased TGF-β1–Smad signaling and reduced PI3K/Akt activity, reflected by decreased Bcl-2, increased caspase-9 and p53, and reduced p-Akt, indicating the existence of an “optimal shear window” [66]. Under low-flow conditions, fluid stimulation upregulates integrin αvβ3 and activates downstream PI3K/Akt, leading to increased MMP-9 expression and enhanced transmigration/invasion; when tissue-engineered bone is introduced into the system, this αvβ3–PI3K/Akt–MMP-9 axis is further amplified, with significantly higher migration rates and MMP-9 levels, suggesting that fluid forces cooperate with bone-associated ECM cues to shape an osteotropic migratory phenotype [66].

Low-level WSS can also promote YAP1 nuclear translocation via the ROCK–LIMK–cofilin pathway and upregulate its migration-related transcriptional targets, accompanied by a rapid increase in MMP-2/MMP-9, indicating activation of ECM-degrading programs and matrix-guided invasion routes [56]. When WSS is further elevated, YAP1-dependent outputs are instead suppressed, again consistent with the intensity-dependent pattern in which low-to-moderate shear promotes migration whereas high shear is inhibitory. In addition, PCa cells can convert low-magnitude shear (WSS ≈ 0.05 dyn·cm^−2^) into pro-migratory signals through the mechanosensitive ion channel Piezo1. Taken together, PCa cells display clearly dose- and context-dependent responses to fluid mechanical cues, and these adaptive changes are likely to contribute to hematogenous and lymphatic dissemination as well as local invasive remodeling.

## 2. Mechanical Adaptation of Prostate Cancer Cells

**Figure 2:**
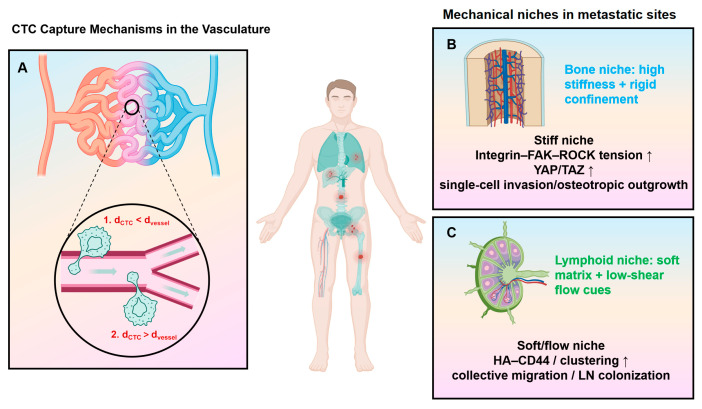
(**A**) CTC capture mechanisms and metastatic dissemination in prostate cancer. Two major routes contribute to the initial binding of CTCs to the vessel wall, a prerequisite for extravasation. Physical trapping occurs when a CTC enters a microvessel with a diameter smaller than the cell (CTC diameter > vessel diameter), a process favored in capillaries (<10 μm) given that epithelial-derived CTCs are typically larger than 10 μm. Adhesive capture predominates in larger vessels (CTC diameter < vessel diameter) and depends on receptor–ligand interactions between tumor cells and the vascular endothelium. (**B**,**C**) Mechanical adaptation of prostate cancer cells during dissemination and in organ-specific mechanical niches. (Created with BioRender.com).

Metastatic dissemination of tumor cells is fundamentally dependent on their ability to adhere to the ECM, within which type I collagen serves as a major scaffold for PCa cell attachment and anchorage. Following this initial adhesion step, cells must migrate: tumor cells degrade collagen through matrix metalloproteinases (MMPs) to break free from the ECM, and then travel toward distant sites under the combined influence of blood flow patterns and chemokine gradients [67,68,69]. As illustrated in Figure 2, once prostate cancer cells enter the circulation, their successful dissemination further depends on mechanical interactions with the vasculature, including physical trapping in narrow capillaries and receptor-mediated adhesive capture in larger vessels, which together facilitate vascular arrest and subsequent extravasation at permissive metastatic sites.

Interestingly, MMP expression peaks in tumors with low to intermediate Gleason scores [2,3,4,5,6,7], but declines in high-grade lesions (Gleason 8–10). This pattern suggests that, at earlier stages, collagen is actively remodeled and degraded in an MMP-dependent manner, whereas in advanced disease, once extensive matrix loss and metastasis have occurred, the demand for MMP secretion diminishes—a phenomenon that may partly account for the limited efficacy of MMP inhibitors in clinical trials [70]. Within the “seed and soil” framework, type I collagen provides a particularly favorable matrix “soil” for PC-3 cells, which readily adhere to collagen I and, in the presence of surface integrins, can establish secondary foci at distant sites. Although adhesion to collagen I can transiently restrain cell detachment and dissemination, from a biomechanical perspective this matrix offers anchorage points that confer a growth advantage within that niche. Much like tree roots penetrating and gripping the soil, where the ground not only supplies nutrients but also tightly encases and stabilizes the trunk against wind and rain, firmly embedded roots require substantial force to uproot. Analogously, once tumor cells form tight adhesive complexes with collagen I, they must generate stronger contractile forces or upregulate matrix-degrading activity to disengage from the ECM and accomplish long-range metastasis.

Recent work further indicates that, as metastatic potential increases, PCa cells display heterogeneous mechanical responses across substrates of different stiffness. Importantly, these stiffness-dependent programs appear to bias distinct migratory strategies in tissue-specific ECM contexts: on stiff matrices that mimic bone, bone-derived metastatic cells tend to rely on nuclear accumulation of YAP/TAZ to enhance single-cell migration and proliferation; by contrast, under softer conditions resembling lymphoid tissue, lymph node–derived cells preferentially upregulate CD44, form multicellular clusters, and spread via collective migration [71]. Conceptually, such plasticity suggests that PCa cells “tune” their mode of movement to the mechanical properties of the surrounding ECM, rather than adopting a single invariant migratory phenotype. These observations support the notion that the mechanical properties of the ECM not only modulate modes of cell movement but may also contribute to the organotropism observed in prostate cancer metastasis [72].

However, reported stiffness–migration relationships are not always consistent across studies. Such discrepancies likely reflect differences in experimental context, including 2D versus 3D architectures [73], ECM composition and ligand density, the degree of confinement, and whether stromal or immune components are present, all of which can reshape adhesion signaling and cytoskeletal programs [74]. These context-dependent outputs underscore the need to consider how mechanical cues are sensed and integrated at the molecular level.

## 3. Mechanical Signal Transduction in Prostate Cancer

**Figure 3:**
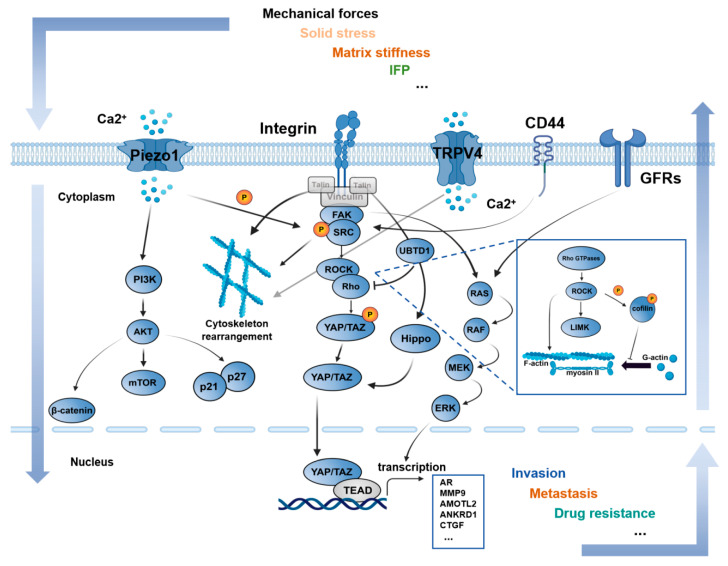
Major mechanotransduction signaling network in prostate cancer. (Created with BioRender.com).

To support the stiffness-dependent phenotypes and context-specific migration programs described above, prostate cancer cells must convert external mechanical cues into intracellular signaling events. Mechanical stimuli within the prostate cancer microenvironment must ultimately be sensed and transduced by tumor cells in order to elicit biological effects. Accumulating evidence indicates that prostate cancer cells are equipped with a range of mechanosensory and mechanotransductive modules capable of converting extracellular physical cues into intracellular signaling and transcriptional responses. In this section, we systematically summarize the major molecular pathways involved in mechanical signal transduction in prostate cancer, with a particular focus on integrin-mediated adhesion signaling, the mechano-transcriptional regulators YAP/TAZ, and the mechanosensitive ion channel Piezo1. It should be noted that only a subset of representative receptors and pathways are discussed in detail here, while additional potential mechanotransductive receptors and modules implicated in prostate cancer are summarized in Table 1. An overview of the major mechanotransduction network and its downstream phenotypic outputs in prostate cancer is provided in Figure 3.

### 3.1. Integrins

Integrins represent one of the major mechanosensory receptors in PCa cells, serving as a physical and signaling interface that couples ECM cues to the actin cytoskeleton and translates mechanical inputs into adhesion- and traction-related intracellular programs [75,76,77]. Upon engagement with ECM ligands, integrin α/β heterodimers undergo conformational activation; their cytoplasmic tails are linked to actin via talin and kindlin, facilitating focal adhesion assembly and maturation. This, in turn, activates the FAK/Src and RhoA/ROCK axes, enhances actomyosin tension, and remodels stress fibers [78,79,80]. When matrix stiffness or local tension increases, integrin clustering and adhesion stabilization are more readily reinforced, thereby amplifying traction output and sustaining migratory persistence. In line with these stiffness-dependent mechanotransduction outputs, high ECM stiffness has been reported to attenuate docetaxel-induced apoptosis and thereby enhance docetaxel resistance in PCa cells, an effect mediated through integrin-associated mechanotransduction signaling [81]. Beyond cell–matrix coupling, integrins may also modulate E-cadherin endocytosis and intercellular junction stability, cooperating with cytoskeletal tension to facilitate transitions between epithelial-like states and more motile single-cell or collective programs.

Beyond serving as core mechanosensory receptors, integrins exert highly context-dependent functions in PCa progression and metastasis. In TRAMP mice, loss of integrin β1 accelerates disease, whereas in vitro evidence indicates that β1 signaling can redirect TGF-β responses toward pro-tumorigenic outputs, underscoring heterodimer-specific and stage-dependent effects [82,83]. Consistently, α11β1 deficiency slows RM11 tumor growth, while α2β1 can restrain proliferation yet promote survival/invasion, and its inhibition reduces EMT programs [84,85]. Integrins also shape tumor–immune interactions: T-cell αVβ8 suppresses antitumor immunity in TRAMPC2 and related models, positioning αVβ8 as a potential immunotherapy target [86], whereas αV expression has been linked to metastatic stem/progenitor-like traits [87]. Additional complexity includes β4-driven expansion of tumor progenitors [88] and the dual role of hemidesmosomal α6β4 as a tumor suppressor in its intact state but pro-oncogenic after hemidesmosome disassembly [89,90]. During dissemination, integrin repertoires differ across metastatic sites (bone/lymph node/liver) [91]; in bone metastasis, enrichment of αV/α5 and contributions of α4/α4β1, α5β1, and α9 support endothelial and stromal interactions [92,93,94,95,96], while αVβ6 and αVβ3 promote osteolytic programs and osteoclastogenesis, respectively [97,98]. Multiple integrins (α6, α2β1, β1) further participate in bone colonization [99,100,101], and β1-dependent cell–cell adhesion can facilitate dormancy escape in bone marrow stromal cultures, implicating integrins in both metastatic seeding and outgrowth [102].

### 3.2. YAP/TAZ

YAP/TAZ are central mechano-transcriptional effectors that enable tumor cells to interpret physical microenvironmental cues. They are highly sensitive to cell crowding/contact inhibition, cell geometry and spreading area, ECM stiffness, and flow-associated shear, and can undergo nuclear translocation and initiate transcriptional programs in either Hippo-dependent or Hippo-independent contexts. Available evidence suggests that YAP activity in PCa correlates with unfavorable clinicopathological features, including higher Gleason grade and enhanced nuclear localization [103,104].

In stiffened matrix settings, increased integrin–focal adhesion–actomyosin coupling can elevate cortical tension via FAK/Src and RhoA–ROCK, promoting YAP/TAZ dephosphorylation and nuclear retention. This may upregulate invasion-associated gene modules such as MMPs and enhance EMT-related programs, while functionally intersecting with proliferative bypass pathways including PI3K/Akt and MAPK [105,106]. Under fluid mechanical cues, low-magnitude WSS can drive Hippo-independent YAP nuclear entry through Piezo1-mediated Ca^2+^ influx and Src activation, and cooperate with ROCK–LIMK–cofilin-dependent cytoskeletal remodeling to augment migration and transwell invasion.

Functionally, YAP and TAZ may display context-biased roles in PCa. YAP appears more directly linked to motility/invasion and metastatic potential, whereas TAZ may preferentially support proliferative programs; flow has been associated with increased TAZ nuclear localization and induction of targets such as AMOTL2, ANKRD1, and CTGF [107]. The ubiquitin-domain protein UBTD1 has been implicated as a potential mechanical brake in this network: its loss can activate RhoA–ROCK, increase cell stiffness and contractility, and reduce YAP ubiquitination (potentially via attenuated interaction with β-TrCP), thereby facilitating YAP activation [108].

Importantly, YAP functionally intersects with androgen receptor (AR) signaling. YAP can act as an AR co-activator in castration-sensitive contexts and remains responsive to androgenic regulation, whereas in CRPC cells it may participate in relatively ligand-independent nuclear complexes [76,109,110]. Thus, YAP/TAZ likely provide a convergence node through which matrix stiffening and fluid stress can reinforce or re-engage AR-driven transcriptional programs, contributing to mechanical adaptation, metastatic progression, and the evolution of therapy resistance.

### 3.3. Piezo1

Piezo1 is a key mechanosensitive ion channel that converts physical cues—particularly shear- and tension-associated inputs—into intracellular Ca^2+^ signals, thereby shaping cellular fate and function [50,111,112,113]. In PCa, multiple studies have reported elevated Piezo1 expression in patient specimens and in cell lines such as PC3 and DU145; Piezo1 knockdown suppresses proliferation and migration in vitro and slows tumor growth in vivo, suggesting a potential pro-tumorigenic and pro-metastatic role that may intersect with enhanced Akt/mTOR activity and cell-cycle–related programs [50,114,115].

Within fluid shear contexts, low-magnitude WSS can activate Piezo1 and trigger Ca^2+^ influx, promote Src Y416 phosphorylation, and induce atypical phosphorylation of YAP at Y357, thereby facilitating YAP/TAZ nuclear retention and transcriptional activation. The pharmacological agonist Yoda1 may synergize with shear stimulation, whereas Piezo1 silencing markedly attenuates shear-induced migration and invasion and reduces metastatic outgrowth in xenograft settings, supporting a central role for the Piezo1–Src–YAP axis in mechanical stress–transcription coupling in PCa [116].

Notably, mechanical signaling may exert context-dependent bidirectional effects. Under certain conditions, shear stress and Piezo1 activation can sensitize tumor cells to TRAIL-induced apoptosis [117], implying that this pathway may not only promote dissemination but could also be therapeutically re-purposed within specific combinatorial settings. Overall, Piezo1 provides an upstream entry point for decoding and amplifying flow-related mechanical cues in PCa and represents a mechanobiology target of translational interest.

**Table 1:** Receptors potentially involved in mechanotransduction in prostate cancer. (Extrapolated: The mechanosensory or mechanotransductive mechanisms of the module are well established in other cancer types or biological systems, but direct mechanical evidence in prostate cancer remains limited; annotations in this table are therefore extrapolated based on mechanistic consistency and reported functional associations in PCa).

Module/Receptor	Category	Evidence Level	Mechanical Modalities	Putative Functional Roles in PCa	Representative References
Integrins	ECM adhesion/mechanosensory receptors	Direct	ECM stiffness, WSS/FSS, tensile load, fiber alignment, cooperates with fluid cues	Link stiffness to FAK/Src signaling; drive adhesion, traction, invasion, and survival.	[118,119,120]
Piezo1	Mechanosensitive ion channel	Direct	ECM stiffness, WSS/FSS, membrane stretch/tension	Shear/tension-sensing Ca^2+^ influx; supports cytoskeletal remodeling and motility.	[114,115,116,117,121]
TRPV4	TRP channel	Extrapolated (limited PCa evidence)	Shear stress, cytoskeleton/junction remodeling, angiogenesis contexts	Shear-induced Ca^2+^ influx; may promote angiogenesis and junction/cytoskeleton remodeling.	[122,123]
TRPC6	TRP channel	Indirect → Direct	Membrane stretch, osmotic/tension changes (often included among shear-related candidates)	Stretch/tension-responsive Ca^2+^ signaling; may enhance migration/invasion.	[124,125,126,127,128,129,130]
TRPM7	TRP channel (kinase-containing)	Indirect → Direct	Fluid shear stress (low-Pa range), mechanical stimulation	Shear/mechanical sensor; regulates adhesion turnover and migration via Ca^2+^/Mg^2+^ signaling.	[131,132,133,134,135]
CD44 (hyaluronan receptor)	HA/glycocalyx–ECM receptor	Extrapolated (limited PCa evidence)	Interstitial flow, convection-associated cues; HA-rich swelling matrix (IFP-related)	Couples HA-rich matrix to motility; linked to collective migration in soft niches.	[136,137,138]
TGF-β/TGF-βR1/2 signaling	Cytokine receptor axis	Extrapolated (limited PCa evidence)	Matrix stiffness, tension, compression/pressure context	Stiffness-amplified pro-fibrotic/EMT signaling; promotes CAF activation and invasion.	[139,140,141,142]
β-catenin	Mechanochemical transcriptional module	Indirect → Direct	Mechanical strain/tension at junctions, stiffness-associated signaling	Tension-sensitive junctional signaling; supports EMT-like states and invasion.	[143,144,145,146]
EGFR (GFR/RTK)	Growth factor receptor (RTK)	Indirect	ECM stiffness, integrin–RTK synergy	Stiffness/integrin synergy sustains proliferation and survival; may enhance invasion.	[147,148,149,150]
MET (HGF receptor; GFR/RTK)	Growth factor receptor (RTK)	Indirect (integrin crosstalk)	HA-rich/ECM remodeling context (mechanochemical coupling)	ECM/integrin crosstalk boosts motility and invasive growth in permissive niches.	[151]
IGF-1R (GFR/RTK)	Growth factor receptor (RTK)	Indirect→ Direct (CD44 cooperation)	Adhesion/tension context modulates RTK persistence/trafficking	Adhesion/tension may prolong signaling; supports survival and therapy tolerance.	[152,153]

## 4. Mechanotransduction–Immunity Crosstalk in Prostate Cancer

Immune cells exhibit pronounced physical characteristics in their life activities. Whether T cells, NK cells, or myeloid cells, their functions rely on sustained and dramatic morphological remodeling, rapid migration through narrow tissue spaces, adhesion and rolling under shear flow, and the formation of dynamic interfaces with tumor cells or antigen-presenting cells (e.g., immune synapses) [154]. Extending this perspective to prostate cancer, tumor-associated mechanical factors (such as changes in matrix stiffness, ECM fibrotic remodeling, and the potential contributions of solid stress/interstitial fluid pressure and fluid-related environments) may collectively shape the physical accessibility of immune cells to tumors and their functional states, thereby constituting “mechanical immune checkpoints” worthy of attention in prostate cancer. In prostate cancer, matrix stiffening has been shown to promote immune evasion through the YAP–TAZ pathway: increased substrate stiffness activates the integrin β1–FAK–YAP axis, upregulates USP8 transcription, thereby stabilizing PD-L1, and accelerates the loss of MHC-I via NBR1-associated selective autophagy, ultimately weakening antitumor immune recognition [155]; these findings also suggest that inhibiting USP8 may serve as a sensitizing strategy for immunotherapy [156]. Meanwhile, the PCa stroma may act as a direct biophysical barrier that restricts T-cell surveillance. In human PCa specimens, collagen VI (Col VI) deposition is associated with the localization of T cells within stromal regions; mechanistic studies further show that purified Col VI markedly suppresses CD4^+^ T-cell motility and reduces actin organization and traction-related features, suggesting that a collagen-rich stroma may cause “T-cell trapping/retention,” thereby reducing effective tumor–immune cell contact [157]. Mechanotransduction in stromal cells may also further influence immune infiltration in prostate cancer. Studies in PCa have defined ECM-associated CAF states that promote collagen deposition and are linked to immune-cold features, and have shown that YAP1 activity regulates CAF states; in vivo, selective depletion of YAP1 in ECM-CAFs increases CD8^+^ T-cell infiltration and activation and enhances anti–PD-1 treatment efficacy [158]. Overall, most current studies support the existence of mechanical immune checkpoints in PCa primarily through immune-phenotype reprogramming, and more PCa models are needed that quantitatively measure solid stress/IFP while assessing immune infiltration and immune effector endpoints, in order to evaluate the feasibility of pressure-targeted mechanical immunomodulatory strategies.

## 5. Diagnosis and Treatment of Prostate Cancer: A Mechanical Perspective

### 5.1. Matrix Softening, Stress Decompression, and Vascular Functional Restoration

Solid stress and interstitial fluid pressure in prostate cancer are thought to arise predominantly from increased ECM crosslinking and an abnormal vascular network that drives leakage and impaired perfusion [54,159]. Accordingly, multiple strategies that aim to reduce matrix stiffness, relieve intratumoral pressure, and improve perfusion may be conceptually translatable to PCa [160]. First, inhibition of LOX family enzymes or MMPs can reduce collagen crosslinking and decrease the Young’s modulus of the matrix, thereby weakening integrin clustering and stress fiber–mediated traction at the source; however, their prostate cancer–specific efficacy in humans still requires prospective validation [161,162,163,164]. Second, targeting proteoglycan and hyaluronan (HA) burden—such as through Hedgehog pathway inhibitors [165,166] or enzymatic degradation of HA [167,168]—has been shown in multiple cancer models to relieve solid stress, reduce IFP, and improve perfusion. In prostate cancer, studies have demonstrated that 4-methylumbelliferone (4-MU) and imatinib can reduce HA accumulation in vivo, potentially by suppressing HA-mediated CD44 activation and downstream PI3K-Akt, and ERK signaling, thereby attenuating cancer cell migration and invasion [169,170]. Third, RAS inhibitors such as losartan can suppress profibrotic signaling pathways, including TGF-β, leading to reduced collagen and HA deposition, vascular decompression, and improved perfusion and drug delivery. A phase II clinical trial has reported beneficial effects of losartan in pancreatic cancer [171]. Fourth, vascular normalization strategies based on anti-VEGF or anti-angiogenic agents can reduce vascular leakage, lower interstitial fluid pressure, and alleviate abnormal stress distributions associated with fluid viscosity [172,173,174]. Clinical trials combining bevacizumab with chemotherapy have demonstrated reductions in interstitial fluid pressure and plasma VEGF levels in rectal cancer [175]. In glioblastoma patients treated with the pan-VEGF receptor tyrosine kinase inhibitor cediranib, improved tumor perfusion was significantly associated with prolonged overall survival in newly diagnosed GBM [176].

In prostate cancer, overall, it is advisable to combine shear-wave or magnetic resonance elastography–based stratification of matrix stiffness with vascular functional assessments to guide combination therapies, while avoiding excessive anti-angiogenic treatment that could provoke secondary perfusion loss and hypoxia rebound [177].

### 5.2. Traction Unloading at the Level of Adhesion and Contractility

The goal of interventions targeting the adhesion–contractility axis is to reduce cellular traction forces and focal adhesion maturation, thereby interrupting the forward amplification of mechanical signaling. Several integrin inhibitors have already been approved for clinical use, and many others are currently under clinical investigation. In prostate cancer, multiple agents targeting integrins or specific integrin subunits have shown clinical potential [178]. Inhibition of FAK/Src can simultaneously suppress parallel proliferative signaling pathways such as PI3K/Akt and MAPK, and therefore often exhibits the greatest therapeutic value when applied as part of combination strategies in stratified settings [179]. Rho–ROCK inhibitors reduce actomyosin tension and relax stress fibers, but their effects differ across cancer types and may depend on cell type, cellular context, and the associated microenvironment [180,181,182,183,184,185]. It should be emphasized that multiple solid tumor models have demonstrated pronounced plasticity in cancer cell migratory modes: when protease activity or adhesion/contractility pathways are partially inhibited, tumor cells can switch between mesenchymal, amoeboid, or collective migration to maintain invasive capacity [186,187,188]. This phenomenon suggests that long-term, single-axis blockade of adhesion or contractility may theoretically promote a drift of migratory phenotypes toward low-adhesion, highly plastic modes. Although direct evidence for this process in prostate cancer is still limited, the risk of adaptive reprogramming should be fully considered when designing mechanically targeted therapies.

Therefore, such strategies may be more appropriately combined with matrix-softening approaches or YAP/TEAD pathway inhibition, with circulating tumor cell (CTC) dynamics, metastatic events, and functional imaging serving as surrogate endpoints aligned with their “anti-invasion/traction-reducing” mechanistic properties.

### 5.3. Blockade of the Mechanosensing Cascade

At the level of mechanosensing, mechanosensitive modules such as Piezo1, the CD44– HA axis, and selected TRP channels participate in detecting extrinsic physical cues and translating them into intracellular signals. At the transcriptional level, YAP/TAZ–TEAD integrates upstream inputs from integrin/FAK/Src–ROCK signaling into defined gene programs and functionally intertwines with AR signaling. For PCa patients who may exhibit a high circulating tumor cell burden, lymph node–predominant early dissemination, or pronounced fluid mechanics–driven features, a mechanistic rationale supports prioritizing interventions at the mechanosensitive channel level and combining them with adhesion- and transcription-focused strategies to achieve potentially complementary inhibition.

In parallel, inhibition of YAP–TEAD interactions or blockade of YAP/TAZ nuclear translocation may suppress invasive, epithelial–mesenchymal transition (EMT), and remodeling programs elicited by matrix stiffening and fluid shear, while attenuating YAP-mediated co-activation of AR. When combined with androgen deprivation therapy (ADT) or AR signaling inhibitors (ARSIs), such approaches may provide complementary suppression in the context of castration-resistant prostate cancer (CRPC) or elevated YAP nuclear localization, thereby delaying the evolution of resistance-associated phenotypes.

### 5.4. Synergistic Pathways Between Mechanical Interventions and Standard-of-Care Therapies

**Figure 4:**
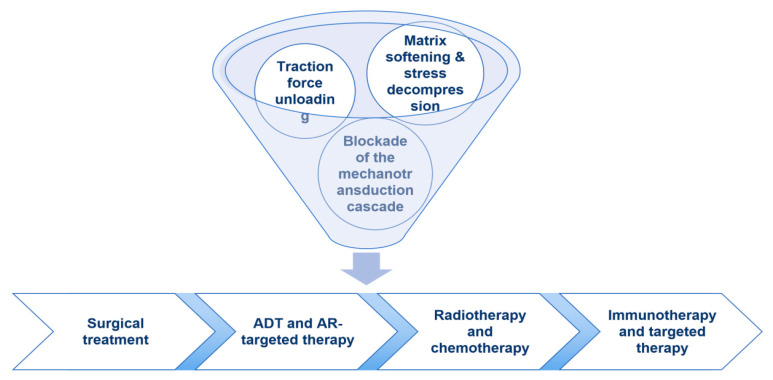
Mechanically informed therapeutic strategies across the continuum of prostate cancer treatment.

Overall, an integrated strategy for combining mechanical interventions with standard-of-care therapies in prostate cancer can be framed around stratification, sequencing, and node-based combinations (Figure 4). Importantly, this framework is hypothesis-generating and is supported mainly by preclinical and cross-tumor evidence; PCa-specific clinical validation remains limited. Accordingly, the following recommendations should be viewed as mechanism-informed concepts whose feasibility and clinical benefit require systematic testing.

From a stratification standpoint, patients who may benefit most could be identified along a putative axis of high traction, increased matrix stiffness, and heightened mechano-transcriptional activity. Candidate markers include integrin signatures (e.g., αvβ3/α2β1), FAK/Src/ROCK activity, YAP/TAZ nuclear localization, and CD44/HA burden, complemented by imaging-based indices such as elastography or shear-wave parameters, DCE-MRI-derived perfusion and permeability readouts, and bone metabolism imaging to estimate metastatic load, thereby improving practical patient selection.

From a sequencing standpoint, a rational yet still unvalidated pathway may begin with matrix and vascular priming (e.g., matrix softening or decompression and vascular normalization) to improve drug delivery and oxygenation, followed by integrin–FAK/Src–ROCK inhibition to blunt traction-driven feed-forward amplification, and then YAP/TEAD inhibition to cap the downstream transcriptional output of mechanotransduction. For patients with hematogenous or lymphatic dissemination tendencies, or with indirect evidence suggesting a role for fluid mechanics in disease progression, Piezo1 inhibition may represent a potential upstream node that complements adhesion- and transcription-level strategies and enables multi-layered synergy. Across these combinations, dosing and safety windows should be carefully considered to avoid excessive loss of mechanical support or perfusion decline driven by overly aggressive anti-angiogenic pressure.

In terms of positioning relative to current standards of care, during the castration-sensitive phase, mild decompression, vascular normalization, and adhesion or traction unloading may serve as a mechanistic sensitization backbone for endocrine therapy and radiotherapy. In the CRPC setting, greater emphasis may be placed on the mechanistic complementarity between YAP/TEAD inhibition and ARSIs, with selective incorporation of integrin, FAK/Src/ROCK, or Piezo1 targeting based on bone-tropic features, matrix stiffness profiles, and the fluid-mechanical context. For patients with a high bone metastatic burden, HA-enriched stroma, and pronounced tissue compression, multi-pathway combinations integrating decompression, anti-adhesion strategies, and AR blockade may jointly improve drug access, reduce traction load, and suppress mechano-transcriptional programs, thereby potentially disrupting the microenvironmental mechanical amplification circuitry that sustains aggressive disease.

At the same time, several key barriers remain for clinical translation: (i) potential on-target toxicity, given the broad physiological roles of mechanotransduction pathways in normal tissues; (ii) heterogeneous and spatially variable drug penetration within stiff or fibrotic lesions; (iii) a lack of standardized, clinically practical biomarkers to quantify tumor mechanics and guide patient stratification; and (iv) uncertainty in defining clinically meaningful endpoints for mechanical modulation (e.g., perfusion or oxygenation changes as surrogate readouts versus long-term disease control). Therefore, systematic validation in PCa-relevant 3D models, immunocompetent in vivo systems, and biomarker-driven early-phase trials will be essential.

## 6. Conclusions

This review systematically summarizes the key mechanical features of PCa and the associated mechanotransduction pathways, with the aim of providing a conceptual framework for mechanics-oriented diagnostic and therapeutic strategies. However, despite rapid progress in recent years, several persistent limitations continue to undermine cross-study comparability, reproducibility, and clinical translatability. First, direct and standardized quantification of core mechanical variables in PCa—including solid stress, interstitial fluid pressure, and shear stress—remains limited. Substantial heterogeneity across studies in model systems, ECM composition, measurement platforms, and parameter definitions makes it difficult to draw consistent conclusions and raises concerns about reproducibility. Second, a large proportion of existing evidence is still derived from 2D culture or simplified in vitro systems, which do not adequately recapitulate prostate-specific mechanical constraints (such as capsular confinement), CAF-driven fibrotic remodeling, abnormal vascular perfusion, or the heterogeneous mechanical niches encountered in bone and lymphatic metastatic environments. Consequently, many reported links between mechanical cues, signaling pathways, and cellular phenotypes remain largely correlative, and rigorous in vivo causal validation is still insufficient.

From a methodological perspective, mechanical effects are often best understood first in controlled in vitro systems, where forces can be imposed and measured more readily. Yet extending mechanobiology into more complex 3D settings and in vivo contexts will require further development of tools that enable precise physical measurements in living cells. Several directions are immediately actionable: prioritizing PCa-relevant standardized 3D platforms (patient-derived organoids, tunable hydrogels, and microfluidic systems) and coupling them with clinically aligned quantitative readouts (such as elastography-based stiffness stratification, vascular functional imaging, and model-assisted inference of pressure and transport constraints). With respect to measurement technologies, the recent emergence of fluorescent tension gauge tools (e.g., TGT probes) offers a potential route toward quantitative assessment of molecular-scale traction forces [189,190]. It will be particularly important to evaluate whether such probes can be incorporated into 3D culture systems and organoid preparations with spatiotemporally resolved readouts. In parallel, genetically encoded tension sensor modules have been developed by inserting a stretchable force-sensing domain between Förster resonance energy transfer (FRET) donor and acceptor pairs [191,192]. When integrated into cytoskeletal and adhesion proteins, these constructs allow dynamic measurements of cytoskeletal tension in living cells. This is conceptually exciting because, in principle, such sensors could be extended to in vivo systems. At present, however, their measurable force range remains limited and applications are confined to a relatively narrow set of contexts. New probes targeting additional physical variables (for example, membrane tension) and improved quantitative frameworks will be critical for further progress. Ultimately, the field remains constrained by a straightforward but consequential principle: we can only interrogate what we can measure.

In addition, recent advances in cancer immunotherapy have increasingly incorporated perspectives of mechanosensing and mechanical regulation, offering new avenues to address therapeutic bottlenecks in PCa. The emerging concept of mechanical immune checkpoints suggests that the mechanical environment may gate immune-cell access and function. Although combining mechanical modulation with established immunotherapies, such as immune checkpoint blockade and CAR-T cell therapy, is an attractive translational possibility, the overall evidence base remains exploratory and will require systematic validation in clinically relevant models and with quantitative endpoints.

Looking forward, progress will depend on moving beyond isolated mechanistic observations toward an integrated research framework that links multiscale quantitative measurements with standardized experimental models and clinically tractable biomarkers, while explicitly incorporating an immune dimension. This includes systematically quantifying how specific mechanical variables shape immune-cell accessibility and functional states, and defining how these effects map onto immunosuppressive pathways. Only through rigorous quantification of key mechanical variables, coherent linkage across biological scales, and alignment with clinically meaningful outcome measures can we more firmly establish the causal role of mechanical forces in PCa progression and evaluate their true feasibility and operability as therapeutic targets.

## Data Availability

No new data were created or analyzed in this study. Data sharing is not applicable to this article.
